# Gliquidone improves retinal injury to relieve diabetic retinopathy via regulation of SIRT1/Notch1 pathway

**DOI:** 10.1186/s12886-021-02215-8

**Published:** 2021-12-27

**Authors:** Mengdan Yu, Lijun Zhang, Shasha Sun, Zhenhua Zhang

**Affiliations:** Department of Ophthalmology, Affiliated Qingdao Central Hospital, Qingdao University, No. 127, Siliu South Road, Qingdao City, 266042 Shandong Province China

**Keywords:** Gliquidone, diabetic retinopathy, inflammation, oxidative stress, SIRT1/Notch1 pathway

## Abstract

**Background:**

Diabetic retinopathy (DR) is a common and potentially devastating microvascular complication of diabetes mellitus (DM). The main features of DR are inflammation and oxidative damage. Gliquidone (GLI) is confirmed to be a hypoglycemic drug by oral administration. The current study is aimed to investigate the role and mechanism of GLI on the pathogenesis of DR.

**Methods:**

High glucose (HG)-induced human retinal endothelial cells (HRECs) were used to explore the anti-inflammatory and anti-oxidant effects of GLI on DR in vitro. Streptozotocin (STZ)-induced DM rats were used to investigate the effects of GLI on retinal structures, inflammation, and oxidative stress. The levels of SIRT1/Notch1 pathway-related proteins were determined by western blotting.

**Results:**

GLI treatment promoted the viability and inhibited the apoptosis of HG-induced HRECs. Meanwhile, the levels of interleukin (IL)-6, IL-1β, tumour necrosis factor alpha and reactive oxygen species were suppressed, while both catalase and superoxide dismutase were elevated after GLI treatment in HG-induced HRECs. Furthermore, we found that Silencing information regulator 2 related enzyme 1 (SIRT1) silencing reversed the inhibiting effects of GLI on the levels of protein Notch1 and effector genes Hes1 and Hey2. Similar anti-inflammatory and anti-oxidant effects of GLI in STZ-induced DM rats were observed. Additionally, GLI administration also repressed vascular hyperpermeability in vivo.

**Conclusion:**

GLI may be an effective agent to improve DR through repression of inflammation and oxidative stress via SIRT1/Notch1 pathway.

## Background

Diabetic retinopathy (DR) is a severe microvascular complication for diabetes mellitus (DM) patients, which is a leading cause of vision impairment for working-age adults worldwide [1, 2]. High level of blood glucose is the main contributing factor to affect DR progression, triggering pathological changes of the retinal cells [3]. In the pathogenesis of DR, endothelial dysfunction, inflammation, and oxidative stress are the three main pathological features [4–6]. Currently, the prevalent treatments for retinal vascular dysfunction such as intraocular injection of anti-vascular endothelial growth factor (VEGF) drugs and steroids have developed rapidly [7–10]. For example, intraocular glucocorticoids are approved for clinical use to treat diabetic macular edema, and topical ocular treatment has been explored with novel selective glucocorticoid receptor agonists [7]. But some adverse effects including retinal neuron destruction, irreversible retinal damage, and even vision loss are still occurred [8–10]. Gliquidone (GLI) is an anti-diabetic medication approved for the clinical use and it is classified as a second-generation sulfonylurea [11–13]. However, researches on the therapeutic efficacy of GLI on DR are relatively rare.

Silent information regulator 2 (SIR2), belonging to the histone deacetylases family, functions as anti-inflammation and oxidation resistance [14]. There are seven homologues of SIR2 named as SIRT1, SIRT2, SIRT3, SIRT4, SIRT5, SIRT6, and SIRT7, respectively. SIRT1 has been confirmed to be deeply involved in the progression of DR [15, 16]. For instance, Jiang et al. found that resveratrol, a specific activator of SIRT1, can remarkably promote the viability of human retinal endothelial cells (HRECs) and repress the secretion of inflammatory cytokines [15]. Ji et al. conducted a DR rat model and demonstrated a decreased expression of SIRT1 in high glucose-induced rat retinal endothelial cells (RRECs) and retinal tissues of DR rats [16]. Meanwhile, they further indicated that overexpressed SIRT1 can accelerate the proliferation of RRECs [16]. Additionally, Notch1 signaling is reported as a downstream effector pathway of SIRT1 to affect the development of human diseases [17–19]. Notch signaling family is a well-known conserved pathway to regulate inflammatory reactions and oxidative stress, [20] and cellular processes such as proliferation, differentiation, and apoptosis [21]. Meanwhile, Dou et al. believed that Notch signaling plays vital roles in retinal angiogenic sprouting/vasculature maturation/vascular stability [22]. As one of the important receptors of Notch, the activation of Notch1 is confirmed to induce vascular permeability, which increases the risk of DR [23]. More importantly, a latest research revealed that SIRT1 can inactivate Notch1 signaling, eventually alleviating the development of sepsis [24]. Tian et al. constructed a diabetic nephropathy (DN) mouse model and showed that GLI may improve oxidative damage and renal interstitial fibrosis to delay the progression of DN through inhibiting Notch1 signaling [25]. However, whether GLI interacts with SIRT1/Notch1 pathway to affect DR progression is still unclear.

In this study, the function of GLI and its relationship with SIRT1/Notch1 pathway in the progression of DR were preliminarily investigated. These results may provide a clinical therapeutic method for DR.

## Methods

### Reagents

Angio-proteomie (Boston, MA, USA) provided human retinal endothelial cells (HRECs). Cell culture and transfection-related reagents were from Invitrogen (Carlsbad, CA, USA). SIRT1-siRNA (si-SIRT1-1/-2) and the negative control (si-NC) were from Sangon Biotech (Shanghai, China). The commercial kits for the measurement of glucose and triglyceride contents were from Boxbio (Beijing, China). The caspase-3 assay kit was from QCbio Science & Technologies (Shanghai, China). Sigma Aldrich (San Luis, MO, USA) provided streptozotocin (STZ) and the commercial kits for the measurement of reactive oxygen species (ROS), catalase (CAT), superoxide dismutase (SOD), and inflammatory cytokines. 3-(4, 5-dimethyl-2-thiazolyl)-2, 5-diphenyl-2-h-tetrazolium bromide (MTT) was from Aladdin (Shanghai, China). Apoptosis detection kit and western blotting analysis-related reagents were from Thermo Fisher Scientific (Waltham, MA, USA). Rat VEGF ELISA kit, the primary antibodies (Notch1, Hes1, Hey2, SIRT1, and GAPDH), and the HRP-conjugated secondary antibody were procured from Abcam (Cambridge, UK).

### DM in vitro model

HRECs were cultured in Dulbecco's modified Eagle's medium containing 10% fatal bovine serum, 1% penicillin/streptomycin at 37°C with 5% CO_2_. Afterwards, HRECs were incubated with normal glucose (NG; 5 mmol/L), high glucose (HG; 30 mmol/L), and HG (30 mmol/L) + GLI (1 μg/ml), respectively. Lipofectamine 3000 was used to perform transfection experiments.

### Measurement for the contents of glucose and triglyceride, and glucose uptake

According to the manufacturer’s instructions, the glucose and triglyceride contents in HRECs of each group were measured using the corresponding commercial kits. The results were assessed using a spectrophotometer at 505 nm (glucose) and 420 nm (triglyceride), respectively. In addition, the 2-deoxy-D-glucose-6-phosphate (2DG6P) uptake assay was performed in strict accordance with the previous study [26].

### Cell viability assay

HRECs (10000 per well) were seeded in 96-well plates for 24 h at 37°C, 5% CO_2_, followed by adding MTT (5 mg/ml) to each well. After incubation for 4 h, the absorbance at 450 nm was measured.

### Caspase-3 activity assay

The activity of caspase-3 in HRECs was assessed using a caspase-3 assay kit under a micro-plate reader with the absorbance of 405 nm.

### Cell apoptosis assay

Cell apoptosis was assessed by flow cytometric analysis. In brief, cells (1 × 10^5^ cells/mL) were cultured in 96-well plates for 24 h, and then stained with V-FITC and PI using an apoptosis detection kit at 25°C for 20 min in the dark. The apoptotic cells were measured using a flow cytometer (BD Biosciences).

### Animal model

All animal experiments in this study were in strict accordance with the protocols stated in the Guide for the Care and Use of Laboratory Animals and approved by the ethical committee of Affiliated Qingdao Central Hospital, Qingdao University. A total of 24 male Sprague Dawley (SD) rats (200-220 g, 10 weeks) purchased from EseBio (Shanghai, China) was allowed to adapt to the laboratory environment before testing. One week later, the rats were assigned into three groups (n = 8): the normal group, the DM group, and the DM + GLI group. STZ (60 mg/kg body weight) dissolved in citrate buffer (10 mM) was intraperitoneally (i.p.) injected into rats to induce DM, while the rats injected with equal volume of citrate buffer were served as the normal group. The level of fasting blood glucose that more than 16.7 mmol/L was served as the DM rats. Afterwards, GLI (50 mg/kg body weight per day) dissolved in dimethylformamide (DMF) was administrated to rats in the DM + GLI group lasting for 12 weeks by intragastric administration [13]. The rats in the normal and DM groups were administrated with equal volume of DMF.

### Assessment for retinal vascular permeability

After GLI administration, rats in each group were anesthetized by pentobarbital sodium (200 mg/kg; i.p.) and then administered with Evans blue dye (30 mg/kg) via femoral vein injection. Subsequently, phosphate buffer saline was perfused into the left ventricle of rats and then 4% paraformaldehyde. The retinal tissues were then collected. Evans blue dye was extracted in formamide and quantified by absorbance measurement at 420 nm.

### Analysis for inflammation, oxidative stress and VEGF level

According to the manufacturer’s instructions, the levels of interleukin (IL)-6, IL-1β, tumour necrosis factor alpha (TNF-)α, ROS, CAT, SOD, and VEGF in retina tissues of DM rats and/or HG-induced HRECs were measured by the corresponding commercial kits.

### VEGF mRNA level measurement

The mRNA level of VEGF was detected by quantitative real time PCR (qRT-PCR). In brief, total RNA extracted from retinal tissues of DM rats was used for cDNA synthesis and then for qRT-PCR analysis. The 2^-ΔΔCt^ method was utilized to calculate the VEGF mRNA level and GAPDH was used as the internal control.

### Hematoxylin-eosin (HE) staining

The retinal tissues were fixed in 4% paraformaldehyde for 24 h, followed by embedding in paraffin sectioned at 5 μm thickness. All the sections were stained with H&E staining immediately and then were observed by a light microscopy.

### Western blot

Proteins were extracted from HG-induced HRECs and retinal tissues of DM rats using RIPA lysis buffer. The protein concentrations were then determined using a BCA kit. Then proteins were analyzed by 10% polyacrylamide gel electrophoresis, and transferred to PVDF membranes. Before the primary antibodies incubation, the membrane was blocked using 5% nonfat milk. Then the membrane was incubated with the secondary antibody for 1 h, and analyzed by ECL kit. GAPDH was internal reference of Notch1, Hes1, Hey2, and SIRT1.

### Statistical analysis

The data were analyzed using SPSS 22.0 software and expressed in the form of mean ± SD. Student's t-test and one-way ANOVA followed by Tukey's multiple comparisons test were used for data analysis. *P* values < 0.05 were considered statistically significant.

## Results

### HG treatment increases the contents of glucose, triglyceride, and 2DG6P in HRECs

**Fig. 1:**
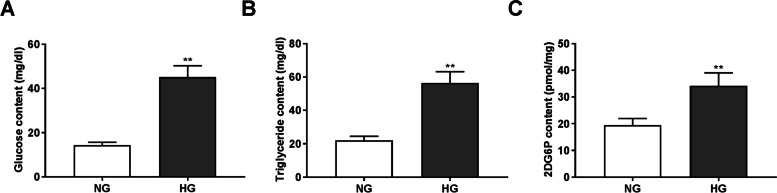
HG treatment increases the contents of glucose, triglyceride, and 2DG6P in HRECs. (**A**) The content of glucose in HRECs. (**B**) The content of triglyceride in HRECs. (**C**) The glucose uptake in HRECs. **P < 0.01 vs. the NG group. HG: 30 mmol/L glucose. NG: 5 mmol/L glucose

The contents of glucose, triglyceride, and 2DG6P in HG-induced HRECs were measured. As illustrated in Fig. 1A-C, we found that compared to the NG groups, HG treatment significantly increased the contents of glucose, triglyceride, and 2DG6P in HRECs (*P* < 0.01), suggesting that a DM cell model was successfully established.

### GLI promotes cell viability but inhibits the apoptosis of HG-induced HRECs

**Fig. 2:**
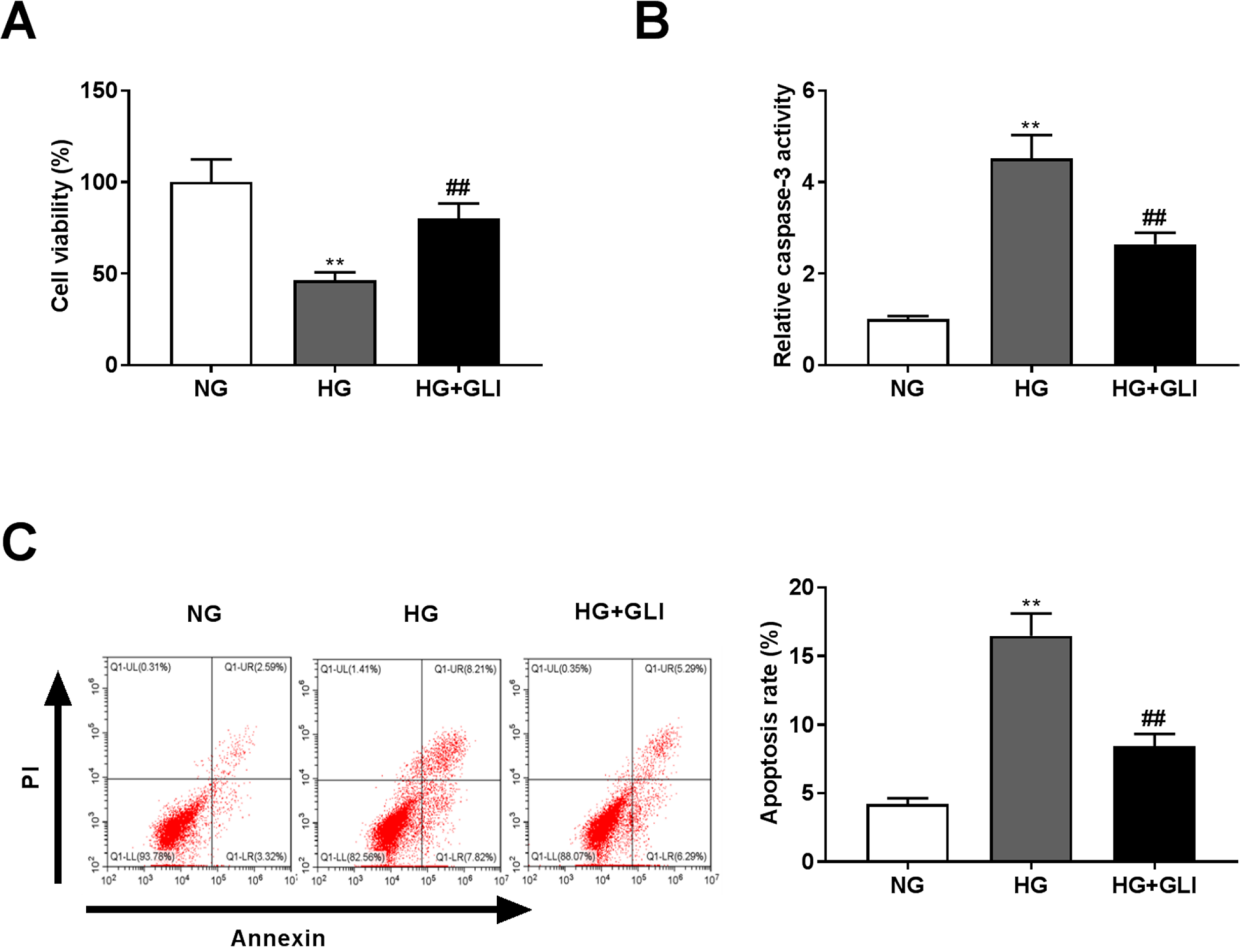
GLI promotes cell viability but inhibits the apoptosis in HG-induced HRECs. (**A**) The viability of HG-induced HRECs was measured by MTT assay. (**B**) Caspase-3 activity in HG-induced HRECs. (**C**) The apoptosis of HG-induced HRECs was assessed by flow cytometric analysis. **P < 0.01 vs. the NG group. ^##^*P* < 0.01 vs. the HG group. HG: 30 mmol/L glucose. NG: 5 mmol/L glucose. GLI: 1 μg/ml gliquidone

The influences of GLI on HREC viability and apoptosis under HG conditions were then explored. We demonstrated that in HRECs, HG treatment remarkably decreased the cell viability, but promoted caspase-3 activity and apoptosis (Fig. 2A-C, *P* < 0.01). After addition of GLI, HG-induced HRECs showed the increased cell viability and the reduced caspase-3 activity and apoptosis rate (*P* < 0.01).

### GLI suppresses the inflammatory responses and oxidative stress caused by HG treatment in HRECs

**Fig. 3:**
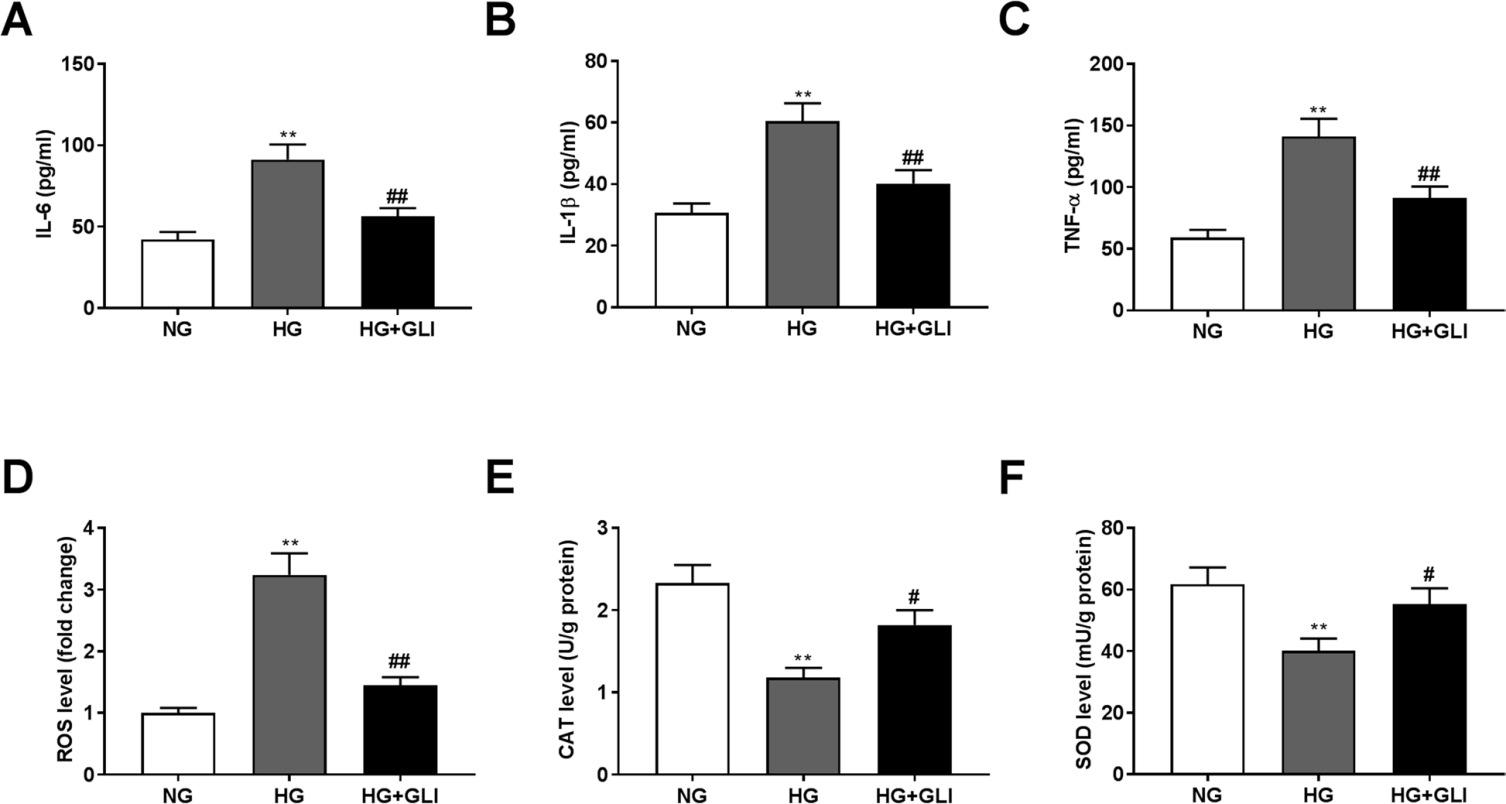
GLI suppresses the inflammatory responses and oxidative stress caused by HG in HRECs. (**A**) The level of IL-6 in HG-induced HRECs was measured by ELISA assay. (**B**) The level of IL-β in HG-induced HRECs was measured by ELISA assay. (**C**) The level of TNF-α in HG-induced HRECs was measured by ELISA assay. (**D**) The level of ROD in HG-induced HRECs was measured by a commercial kit. (**E**) The level of CAT in HG-induced HRECs was measured by a commercial kit. (**F**) The level of SOD in HG-induced HRECs was measured by a commercial kit. **P < 0.01 vs. the NG group. ^#^*P* < 0.05, ^##^*P* < 0.01 vs. the HG group. HG: 30 mmol/L glucose. NG: 5 mmol/L glucose. GLI: 1 μg/ml gliquidone

Both inflammation and oxidative damage are confirmed to affect DR progression [27, 28]. Therefore, the effects of GLI on inflammatory cytokines (IL-6, IL-1β, and TNF-α) and oxidative stress-related factors (ROS, CAT, and SOD) in HG-induced HRECs were further investigated. As expected, HG treatment significantly elevated the levels of IL-6, IL-1β, TNF-α and ROS, and repressed the release of CAT and SOD (Fig. 3A-F, *P* < 0.01). However, these situations were all reversed by GLI treatment (*P* < 0.05).

### GLI represses Notch1 signaling in HG-induced HRECs

**Fig. 4:**
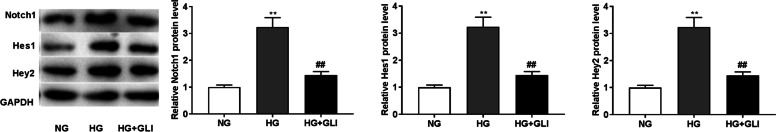
GLI inactivates Notch1 signaling in HG-induced HRECs. The protein levels of Notch1, Hes1, and Hey2 in HG-induced HRECs. **P < 0.01 vs. the NG group. ^##^*P* < 0.01 vs. the HG group. HG: 30 mmol/L glucose. NG: 5 mmol/L glucose. GLI: 1 μg/ml gliquidone

Numerous studies have reported that Notch1 signaling is deeply involved in the pathogenesis of DR via regulation of inflammation and vascular permeability [23, 29]. The interactions between GLI and Notch1 signaling-related genes were then studied. Western blotting analysis showed that the protein levels of Notch1, Hes1 and Hey2 were significantly increased in the HG group relative to the NG group (Fig. 4, *P* < 0.01). Addition of GLI reversed the promoting effects of HG treatment on the levels of these proteins (*P* < 0.01).

### GLI interacts with Notch1/SIRT1 pathway in HG-induced HRECs

**Fig. 5:**
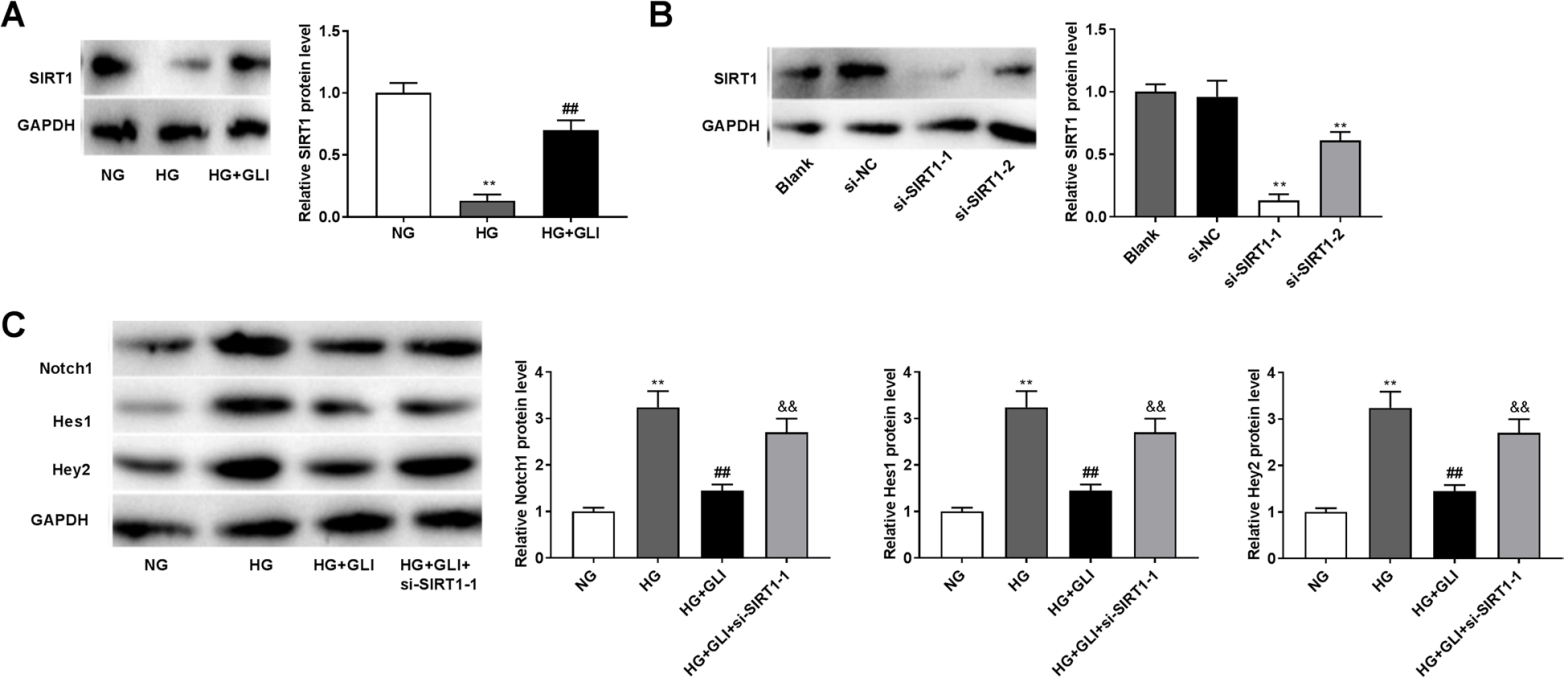
GLI interacts with Notch1/SIRT1 pathway in HG-induced HRECs. (**A**) The protein level of SIRT1 in HG-induced HRECs was determined by western blotting. **P < 0.01 vs. the NG group. ^##^*P* < 0.01 vs. the HG group. (**B**) The protein level of SIRT1 after transfection of si-SIRT1-1/-2/NC was determined by western blotting. **P < 0.01 vs. the si-NC group. (**C**) The protein levels of Notch1, Hes1, and Hey2 in HG-induced HRECs transfected with si-SIRT1-1. **P < 0.01 vs. the NG group. ^##^*P* < 0.01 vs. the HG group. ^&&^*P* < 0.01 vs. the HG + GLI group. HG: 30 mmol/L glucose. NG: 5 mmol/L glucose. GLI: 1 μg/ml gliquidone

The inhibiting effect of SIRT1 on Notch-mediated transcription has been confirmed in renal injured endothelial cells [30]. As presented in Fig. 5A, we indicated that the protein level of SIRT1 was remarkably downregulated in the HG group compared to that in NG group (*P* < 0.01). Meanwhile, GLI eliminated the suppressive effect of HG on SIRT1 protein level (*P* < 0.01). Then, si-SIRT1-1/-2 was transfected into HRECs to determine the transfection efficiency. Western blotting revealed that SIRT1 protein level was significantly reduced in HRECs after transfection (Fig. 5B, *P* < 0.01). We chose si-SIRT1-1 for the subsequent experiments due to the relatively high transfection efficiency. As expected, transfection of si-SIRT1-1 partly eliminated the inhibitory effects of GLI on the levels of Notch1, Hes1, and Hey2 in HG-induced HRECs (Fig. 5C, *P* < 0.01).

### GLI administration attenuates retinal injury in STZ-induced DM rats

**Fig. 6:**
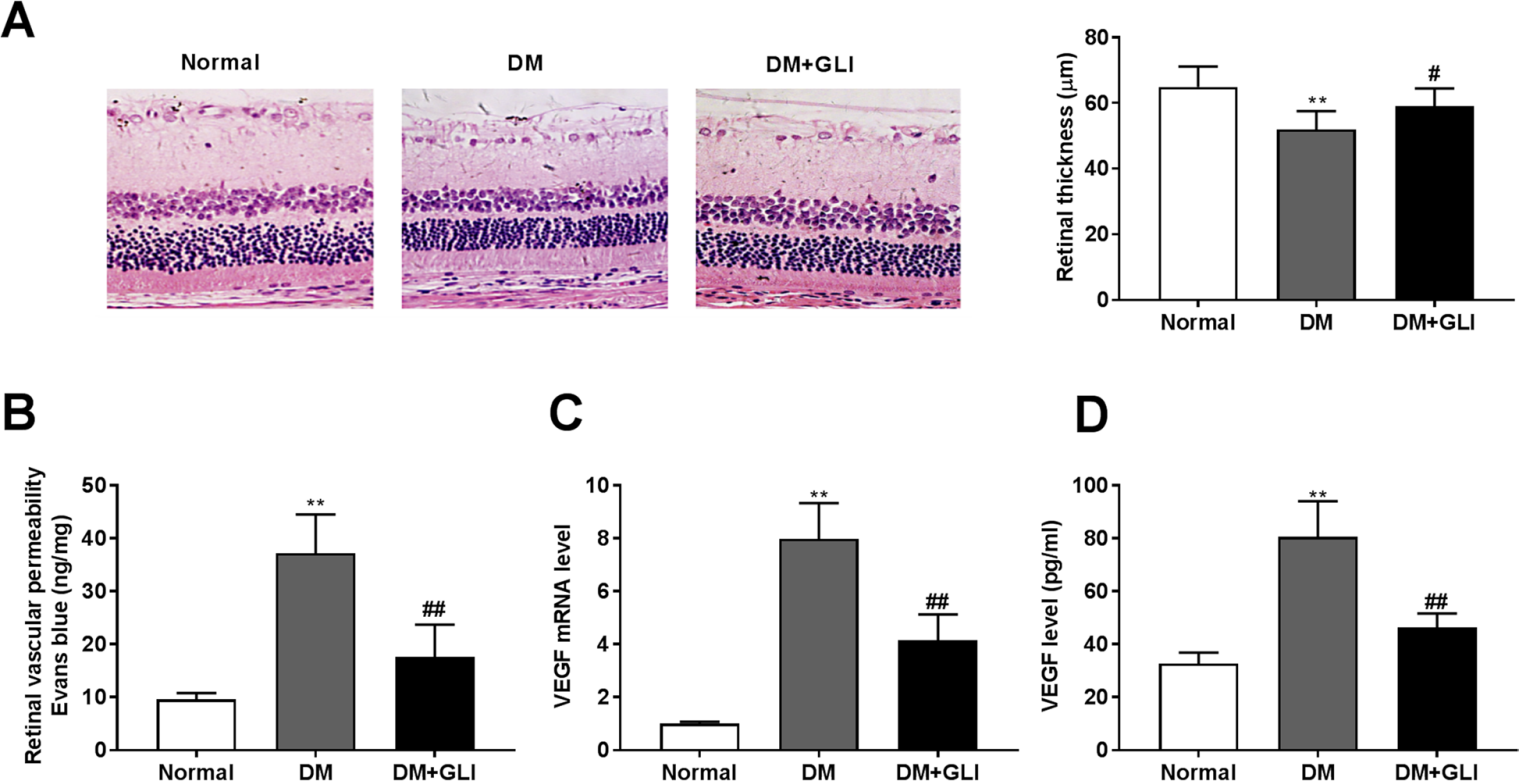
fiGLI administration relieves retinal injury in STZ-induced DM rats. (**A**) Effects of GLI on histology of retina and retina thickness. (**B**) Effects of GLI on vascular permeability in retina of DM rats. (**C**) Effects of GLI on mRNA level of VEGF in retina of DM rats. (**D**) Effects of GLI on protein level of VEGF in retina of DM rats. **P < 0.01 vs. the normal group. ^#^*P* < 0.05, ^##^*P* < 0.01 vs. the DM group. GLI: 50 mg/kg body weight gliquidone

A DM rat model was established to further explore the therapeutic effect of GLI on DR in vivo. The normal group showed the intact retinal structure. A decrease in retinal thickness was found in rats of the DM group (Fig. 6A, *P* < 0.01). However, GLI administration improved the morphological changes in the retinas (*P* < 0.05). The increased vascular permeability can result in retinopathy and vision loss [31]. The Evans blue staining assay was used to assess the retinal vascular permeability in STZ-induced DM rats. We found a significant increase in the retinal vascular permeability of DM rats (Fig. 6B, *P* < 0.01). As expected, after administration of GLI, the retinal vascular permeability was sharply decreased (*P* < 0.01). As previously described, VEGF plays important role in retinal vascular permeability [32]. Therefore, the level of VEGF was then detected. As shown in Fig. 6C-D, both the mRNA expression and protein level of VEGF were elevated in the DM group (*P* < 0.01) but were reduced in the DM + GLI group (*P* < 0.01).

### GLI inhibits inflammation and oxidative injury in retinal tissues of DM rats via SIRT1/Notch1 pathway

**Fig. 7:**
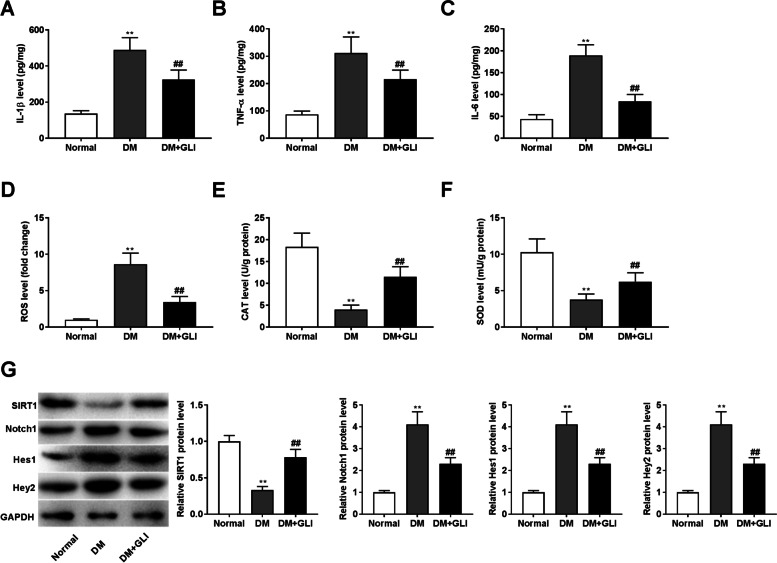
GLI represses inflammation and oxidative injury in retinal tissues of DM rats via SIRT1/Notch1 pathway. (**A**) The level of IL-β in retina of DM rat after GLI administration was measured by ELISA assay. (**B**) The level of TNF-α in retina of DM rat after GLI administration was measured by ELISA assay. (**C**) The level of IL-6 in retina of DM rat after GLI administration was measured by ELISA assay. (**D**) The level of ROS in retina of DM rat after GLI administration was measured by a commercial kit. (**E**) The level of CAT in retina of DM rat after GLI administration was measured by a commercial kit. (**F**) The level of SOD in retina of DM rat after GLI administration was measured by a commercial kit. (**G**) The protein levels of SIRT1, Notch1, Hes1, and Hey2 in retina of DM rat after GLI administration was determined by western blotting. **P < 0.01 vs. the normal group. ^##^*P* < 0.01 vs. the DM group. GLI: 50 mg/kg body weight gliquidone

The effects of GLI administration on inflammation and oxidative injury in retinal tissues of DM rats were also explored. Similar to the results of in vitro model, DM rats showed relatively high levels of IL-1β, TNF-α, IL-6 and ROS, and decreased levels of CAT and SOD (Fig. 7A-F, *P* < 0.01). GLI administration partly improved these adverse impacts (*P* < 0.01). In addition, we also found that compared to the normal group, rats in the DM group had decreased protein level of SIRT1, and increased Notch1, Hes1 and Hey2 protein levels (Fig. 7G, *P* < 0.01). GLI administration also reversed the effects of DM on the levels of these proteins (*P* < 0.01).

## Discussion

More than 80% DM patients may suffer from DR at the first two decades of diabetes [34]. Although the management opinions for DR have greatly improved, some severe side effects are still confirmed to affect the therapeutic efficacy [8–10]. Exploring effective drugs to furthest decrease the side effects in the treatment of DR is extremely urgent. GLI is a well-known hypoglycemic agent in clinic [11]. Previous study has revealed the therapeutic effect of GLI on diabetic nephropathy [25]. But there still no researches on the possible function of GLI on DR progression. This study for the first time uncovers that GLI can relieve DR through regulation of endothelial dysfunction, inflammation, and oxidative stress via SIRT1/Notch1 pathway, suggesting that GLI may be an effective agent for the treatment of DR in clinic.

HG-induced HRECs are generally used as an in vitro model of DR due to the crucial role in angiogenesis [29, 35, 36]. In this study, HG treatment significantly increased the contents of glucose, triglyceride and 2DG6P in HRECs, which is consistent with the data of Long et al. [36]. The results suggested that DR cell model was established successfully. The pathological feature of DR is not only manifested as the increased level of blood glucose, but also the selective apoptosis of endothelial cells [37]. Similarly, we demonstrated that in HG-induced HRECs, the cell viability was decreased, while the apoptosis was promoted. Addition of GLI promoted cell viability and repressed apoptosis of HG-induced HRECs. The experiments indicated that GLI treatment can effectively inhibit the development of DR in a cellular level. Meanwhile, our in vivo experiments that GLI administration improved the decreased retinal thickness caused by DM. Interestingly, Zhu et al. investigated the effect of TGR5 receptor agonist (INT-777) on morphology of retinal tissues in a STZ-induced rat model and found a decrease in retinal thickness induced by diabetes, while intravitreal injection of INT-777 alleviated such morphological changes in the retinas, suggesting that TGR5 agonism is protective against diabetes-induced retinal vascular injury [38]. As a result, we also believed that GLI can alleviate retinal vascular injury in the progression of DR. As well accepted, excessive inflammation responses and oxidative damage are exited in the progression of DR [27, 28]. In the current study, we found enhanced inflammatory reactions and oxidative injuries in HG-induced HRECs and STZ-induced DM rats. In addition, we also demonstrated inflammatory reactions and oxidative stress were relieved by GLI administration both in vitro and in vivo. Similarly, Li et al. focused on the protective effects of sulforaphane on inflammation and peroxidation both in vitro and in vivo, and demonstrated that sulforaphane can attenuate the development of DR through inhibiting inflammation and oxidative stress in HG-induced rat Müller cells and STZ-induced DM rats [39]. Combined the previous results, we concluded that GLI may be also an effective agent to repress inflammation and oxidative stress in DR progression. Vascular hyperpermeability is the initial pathologic feature in DR capillaries, [40] and the increased level of VEGF is confirmed to be closely associated with retinal vascular permeability [32]. Previous researches have confirmed that some agents such as blueberry anthocyanins [41], resveratrol [42], and melatonin [43] can modulate vascular permeability via suppression of VEGF level in DR rats. In this investigation, as expected, DM rats showed relatively high level of VEGF, while administration of GLI repressed the release of VEGF. We speculated that GLI may be helpful for the inhibition of vascular hyperpermeability in the progression of DR. Based on the above data, we believed that GLI may be also an effective agent to ameliorate DR through suppressing a series of pathological processes of DR including endothelial cell apoptosis, inflammation, oxidative damage, and vascular hyperpermeability. In clinic, GLI may inhibit endothelial dysfunction, inflammation responses, oxidative injuries and vascular hyperpermeability, thereby contributing to the improvement of poor prognosis of DR patients.

Notch1 signaling and the corresponding effector genes act as important roles in prevention of inflammation and oxidative stress in numerous human diseases, such as arthritis, [44] enteritis, [45] myocardial injury, [46] and chronic obstructive pulmonary disease [47]. We speculated that there may be some relationships between GLI and Notch1 signaling. As expected, the in vitro and in vivo experiments showed that the protein levels of Notch1 and effector genes (Hes1 and Hey2) were elevated in HG-induced HRECs and STZ-induced DM rats, which suggested that the activation Notch1 signaling may accelerate the development of DR. Similarly, Miloudi et al. believed that the activation of Notch1 increases the risk of DR via inducing vascular permeability [23]. Additionally, we further indicated that GLI partly eliminated the promoting effects of HG on Notch1 signaling-related proteins. The results implied that GLI can repress the activation of Notch1 signaling. Notch1 signaling has been reported as a downstream effector pathway of SIRT1 to affect disorder progression, such as liver fibrosis, breast cancer, and lung cancer [17–19]. We then further speculated GLI may also interact with SIRT1 in DR progression. Firstly, we found that SIRT1 protein level was downregulated in HG-induced HRECs and DM rats. Similarly, a decreased expression of SIRT1 is found in HG-induced RRECs and retinal tissues of DR rats [16]. However, GLI treatment significantly elevated the protein level of SIRT1, suggesting that GLI may induce the production of SIRT1. Based on the results that GLI can inactivate Notch1 signaling, we suggested that GLI may interact with SIRT1/Notch1 pathway to affect DR progression. As expected, we found that downregulation of SIRT1 partly attenuated the inhibiting effects of GLI on the levels of Notch1 signaling-related proteins. Therefore, the SIRT1/Notch1 signaling may be an important regulatory pathway in DR, providing a direction for the research of novel targeted drugs in clinic. Assuredly, except for SIRT1/Notch1 pathway, there are numerous signaling pathways involved in DR progression, such as PlGF/ERK [48], PLA2/COX-2/VEGF-A [49], TLR4/NF-kappaB [50], and PI3K/Akt/mTOR [51]. We speculated GLI may also ameliorate DR via these pathways. This is may be a limitation of this study and we will elucidate these issues in future studies. Additionally, some other factors have been linked to the progression of DR such as miRNAs [52, 53] and lncRNAs [54, 55]. The interaction of GLI with them should be further explored.

## Conclusions

In summary, the present investigation indicates that GLI may be an effective agent to improve DR through inhibiting inflammation and oxidative stress via SIRT1/Notch1 pathway. Our findings clarify the action mechanism of GLI on the development of DR, and may provide an effective drug for DR therapy in clinic.

## Data Availability

The datasets used and/or analysed during the current study are available from the corresponding author on reasonable request.

## Abbreviations

DR: Diabetic retinopathy
DM: diabetes mellitus
GLI: Gliquidone
HG: High glucose
HRECs: human retinal endothelial cells
STZ: Streptozotocin
